# Poncet disease in a patient with smear-negative pulmonary tuberculosis: A case report

**DOI:** 10.51866/cr.581

**Published:** 2024-08-18

**Authors:** Poh Siang Ooi, Hartini Ismail, Gayatherri Meganathan, Nurfathehatul Nabila Saidi, Yeogeashweary Dhamotharan, Hui Heng Chua

**Affiliations:** 1 MD, MMed (Family Medicine), Klinik Kesihatan Kepala Batas, Lot 1466, Mukim 6, Kepala Batas, Penang, Malaysia. Email: ooipohsiang@gmail.com; 2 MBBS, Klinik Kesihatan Kepala Batas, Lot 1466, Mukim 6, Kepala Batas, Ministry of Health Malaysia, Penang, Malaysia.; 3 MBBS, Klinik Kesihatan Kepala Batas, Lot 1466, Mukim 6, Kepala Batas, Ministry of Health Malaysia, Penang, Malaysia.; 4 MBBS, Klinik Kesihatan Kepala Batas, Lot 1466, Mukim 6, Kepala Batas, Ministry of Health Malaysia, Penang, Malaysia.; 5 MBBS, Klinik Kesihatan Kepala Batas, Lot 1466, Mukim 6, Kepala Batas, Ministry of Health Malaysia, Penang, Malaysia.; 6 MD, MPath (Anatomic Pathology), Hospital Pulau Pinang, Jalan Residensi, George Town, Ministry of Health Malaysia, Penang, Malaysia.

**Keywords:** Smear-negative pulmonary tuberculosis, Poncet disease, Joint pain, Erythema nodosum

## Abstract

Pulmonary tuberculosis poses a diagnostic dilemma to clinicians especially in the absence of typical presentation. The hypersensitivity to tuberculosis infection in other parts of the body can lead to nondestructive, para-infectious arthritis. This is known as Poncet disease, one of the clinical syndromes of musculoskeletal tuberculosis. Herein, we report a case of smear-negative pulmonary tuberculosis presenting with atypical features. It started with multiple joint pain, followed by the presence of multiple tender nodular skin lesions over the bilateral shins and wrist. Subsequent investigations led to the diagnosis of smear-negative pulmonary tuberculosis. Joint pain and erythema nodosum disappeared soon after antituberculosis therapy, supporting the diagnosis of Poncet disease.

## Introduction

Tuberculosis was the world’s second leading cause of death attributed to infectious diseases in 2022 after COVID-19. More than 10 million people are diagnosed with tuberculosis every year. The disease typically affects the lungs (pulmonary tuberculosis) but can also affect other sites.^1^ Pulmonary tuberculosis can be further divided into smear-positive and smear- negative types depending on the sputum smear result. The hypersensitivity to tuberculosis infection in other parts of the body leading to non-destructive, para-infectious arthritis is known as Poncet disease.^2^ Herein, we present a case of polyarthritis and erythema nodosum in which Poncet disease was diagnosed after the symptoms resolved with the initiation of therapy for smear-negative pulmonary tuberculosis.

## Case presentation

A 30-year-old woman, Madam R, presented with multiple joint pain over 2 months. The affected joints included the bilateral knees, ankles, feet, wrists, left proximal interphalangeal joint of the middle finger and right metacarpal phalangeal joint of the index finger. She needed analgesia because her pain score was around 8. The joint pain was associated with swelling and morning stiffness. She also experienced weight loss of 6 kg in 2 months from 44 to 38 kg. She denied any recent fever or infection. At this time, no rashes were observed. The patient had a fever and cough, which were diagnosed under COVID-19 category 2B 3 months ago, and was therefore quarantined at home. Her symptoms during COVID-19 infection did not include joint pain.

The patient was a physiotherapist, and her joint pain significantly affected her daily activity at work. She was on medical leave frequently due to such pain. Examination of the affected joints showed mildly swollen joints with tenderness. Radiography of the affected joints showed no abnormalities. Chest radiography also did not show any lesions. Her C-reactive protein level was found to be 28.9 mg/L (<5.0), while her rheumatoid factor level was 10 IU/mL (<14). Her American College of Rheumatology score was 7, and she was then referred to a rheumatologist for review for possible seronegative rheumatoid arthritis. The diagnosis after assessment from the rheumatology team was polyarthralgia likely reactive arthritis secondary to viral infection.

The anti-cyclic citrullinated peptide antibody was found to be negative at 5.1 U/mL (<5.25 U/mL). She was treated with NSAIDs for her joint pain.

**Figure 1:**
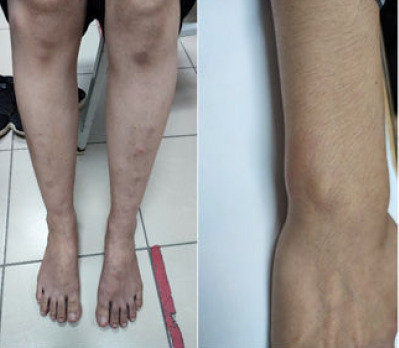
Erythema nodosum over the bilateral shins and right wrist.

The patient returned 2 months later with persistent joint pain along with a new complaint of painful skin nodules over her bilateral shins for 3 weeks (Figure 1). There was a 3-day history of fever with no other notable features within the 3-week period. She did not take any oral contraceptive pills, antibiotics or traditional medication. Her body weight remained consistent at around 38 kg. There were no other pulmonary tuberculosis symptoms such as prolonged cough or haemoptysis. There was also no history of tuberculosis contact. Examination of her shins revealed multiple nodular lesions that were sized <1 cm, mildly erythematous and tender on palpation. A similar lesion was also found on the right wrist (Figure 1). The case was referred to a dermatologist as erythema nodosum for further assessment. A skin biopsy sample was taken from her right shin, and histopathology examination revealed features consistent with erythema nodosum. Periodic acid-Schiff stain for fungal organisms and Ziehl-Neelsen stain for acid-fast bacilli were negative. Additional blood tests were performed to determine the cause of erythema nodosum. Antinuclear antibody was negative; the erythrocyte sedimentation rate was 16 mm/h; the white blood cell count was 8.1×10^3^μL; the liver and kidney functions were normal; and *Mycoplasma pneumoniae* antibody, syphilis and antistreptolysin O titre testing yielded negative findings. Only the Mantoux test demonstrated a positive result at 15 mm.

**Figure 2:**
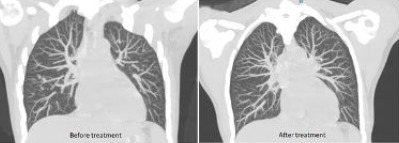
Thoracic high-resolution computed tomography scans before and after antituberculosis therapy.

Given the positive Mantoux test result and the subsequent positive interferon-gamma release assay result, the case was discussed with a chest physician for smear-negative pulmonary tuberculosis. High-resolution computed tomography (HRCT) of the thorax was planned, and it showed multiple solid, ground glass and part-solid nodules in the bilateral lungs. Plate atelectasis with surrounding air trapping and multiple nodules in the tree in bud configuration were noted in the left lower lobe. The features were suggestive of pulmonary tuberculosis (Figure 2). She was then arranged for bronchoscopy. Bronchoalveolar lavage showed negative sputum acid-fast bacillus results, and Xpert MTB/RIF was not detected. There were few pus cells, and the results were negative for both gram-positive and gram-negative cocci. No pathogens were found in the culture. Mycobacterium tuberculosis culture showed no growth after 8 weeks of incubation.

The patient’s condition was then treated as smear-negative pulmonary tuberculosis with a standard antituberculosis regimen involving a 2-month intensive phase with isoniazid, rifampicin, pyrazinamide and ethambutol, followed by a 4-month maintenance phase with isoniazid and rifampicin. Her joint pain and nodular skin lesions disappeared soon after, and she gained weight. Repeated thoracic HRCT also showed improved lung findings compared to the previous examination (Figure 2).

## Discussion

The typical clinical features of active pulmonary tuberculosis in adult patients include productive cough, haemoptysis, loss of appetite, unexplained weight loss, fever, night sweats and fatigue. The symptoms of smear-positive and smear-negative pulmonary tuberculosis can be similar.^3^ A retrospective study found that the absence of cough and the absence of a radiographic pattern typical of tuberculosis were associated with smear-negative tuberculosis.^4^ In the present case, the diagnosis of pulmonary tuberculosis was delayed because Madam R presented only with weight loss, a transient history of fever and negative acid-fast bacillus sputum smears. The appearance of erythema nodosum and the subsequent workup led to the diagnosis of pulmonary tuberculosis based on the positive Mantoux test result.

Diagnosing smear-negative pulmonary tuberculosis is a dilemma for treating physicians. It became even more difficult in this case because Madam R did not exhibit the majority of typical tuberculosis symptoms. Due to the non-specific nature of the symptoms of tuberculosis, a systematic approach should be used to diagnose tuberculosis in patients whose sputum smears are negative. The algorithm for diagnosing smear-negative pulmonary tuberculosis developed by the World Health Organization in 2003 requires at least three negative sputum smears, chest radiography findings consistent with tuberculosis and a lack of response to a trial of broad-spectrum antimicrobial agents.^5^ Two sputum samples from Madam R obtained via direct sputum collection and one sample via bronchoalveolar lavage were negative. Thoracic HRCT showed features that were suggestive of pulmonary tuberculosis. Without trial of any antibiotics, the treating chest physician decided to treat Madam R as having smear-negative pulmonary tuberculosis.

Smear-negative cases account for 30%-60% of all pulmonary tuberculosis cases and 10%- 20% of tuberculosis transmission cases at the population level.^4^ Delays in diagnosing pulmonary tuberculosis are possible if a patient does not present with typical tuberculosis symptoms. Delayed diagnosis or underdiagnosis of tuberculosis can result in more severe consequences, including disability and possibly death, as well as ongoing transmission of the disease.^5^ Atypical presentations of tuberculosis combined with delayed diagnosis contribute to this region’s high incidence of tuberculosis.^6^ Clinicians should keep in mind that patients with tuberculosis may present with extrapulmonary manifestations such as joint pain and erythema nodosum, as shown in this case.^7^ Madam R had reactive arthritis and erythema nodosum without typical tuberculosis symptoms. Poncet disease was diagnosed after the diagnosis of smear-negative pulmonary tuberculosis and a retrospective review of her initial symptoms.

Poncet disease occurs when a patient with active visceral or disseminated tuberculosis develops non-destructive, para-infectious and symmetric polyarthritis. There will be an absence of joint infection by tuberculosis, and it may be accompanied with episcleritis and/or erythema nodosum on the shins, thighs or upper limbs. Poncet disease can resolve completely with antituberculosis therapy.^2^ Sharma and Pinto proposed diagnostic criteria after reviewing 23 case series (Table 1).^8^

**Table 1:** Sharma and Pinto’s diagnostic criteria for Poncet’s arthritis.

Criteria	Description
Essential	Inflammatory, non-erosive, non-deforming arthritis Exclusion of other causes of inflammatory arthritis
Major	Concurrent diagnosis of extra-articular tuberculosis Complete response to antituberculosis therapy
Minor	Mantoux test positivity Associated hypersensitivity phenomenon, such as erythema nodosum, tuberculids or phlyctenular keratoconjunctivitis Absence of sacroiliac and axial involvement
**For diagnosis**	
Definite	Essential + two major
Probable	Essential + one major + three minor
Possible	Essential + one major + two minor or Essential + three minor

Most Poncet disease cases present with oligoarthritis, with the ankle being the most common joint involved, followed by the knee. Small joints such as the wrist and hand joints are less commonly affected.^8^'^9^ The pattern of joint involvement in the present case was polyarticular including both large and small joints, and the diagnosis of rheumatoid arthritis was excluded. Madam R also fulfilled all major and minor diagnostic criteria, as she was diagnosed with smear-negative pulmonary tuberculosis, and her symptoms resolved with antituberculosis therapy. Additionally, her Mantoux test result was positive, and she developed erythema nodosum following joint pain.

The majority of previously reported Poncet disease cases have confirmed tuberculosis infection, either pulmonary or extrapulmonary.^6,8,10^ In contrast, there was no positive culture at the time of diagnosis of the present case. The diagnosis of smear-negative pulmonary tuberculosis in this case was based on the clinical features and radiological findings. There was a total of 8 months from the time Madam R started to experience joint pain to the time she was diagnosed with pulmonary tuberculosis. Nonetheless, Madam R recovered well after the treatment was initiated.

## Conclusion

Poncet disease is one of the clinical syndromes of musculoskeletal tuberculosis.^2^ It is relatively rare even in countries with a high incidence of tuberculosis.^8^ Primary care physicians are frequently the first point of contact for patients with various complaints. A high level of clinical suspicion and detailed physical examination are pivotal for early detection of tuberculosis, particularly cases with atypical presentation.

## References

[1] World Health Organization. (2023.). Global Tuberculosis Report..

[2] Atukorala I, Chang T, Farrar J, Hotez PJ, Junghanss T, Kang G, Lalloo D, White NJ (2014). Manson’s Tropical Infectious Diseases (Twenty-Third Edition)..

[3] Clinical Practice Guidelines Management of Tuberculosis: Malaysian Health Technology Assessment Section (MaHTAS) (2021).

[4] Campos LC, Rocha MV, Willers DM, Silva DR (2016). Characteristics of patients with smear-negative pulmonary tuberculosis (TB) in a region with high TB and HIV prevalence.. PLoS One..

[5] Tuberculosis Coalition for Technical Assistance. (2006). International Standards for Tuberculosis Care (ISTC)..

[6] Irmi Z, Zaiton A, Faezah H (2013). Reactive arthritis in tuberculosis: a case of Poncet’s disease.. Malays Fam Physician..

[7] Kroot E, Hazes J, Colin E, Dolhain R (2007). Poncet’s disease: reactive arthritis accompanying tuberculosis. Two case reports and a review of the literature.. Rheumatology..

[8] Sharma A, Pinto B, Dogra S (2016). A case series and review of Poncet’s disease, and the utility of current diagnostic criteria.. Int J Rheum Dis..

[9] Rueda JC, Crepy MF, Mantilla RD (2013). Clinical features of Poncet’s disease. From the description of 198 cases found in the literature.. Clin Rheumatol..

[10] Fehr A, El-Nouby F, Eltony AA, Abdelkareem Y, Bogdady S (2017). Poncet disease, tuberculosisarthritis: a case report in upper Egypt and a review of the literature.. Egypt Rheumatol Rehabil..

